# Inheritance of physico-chemical properties and ROS generation by carbon quantum dots derived from pyrolytically carbonized bacterial sources

**DOI:** 10.1016/j.mtbio.2021.100151

**Published:** 2021-10-15

**Authors:** Y. Wu, H. Wei, H.C. van der Mei, J. de Vries, H.J. Busscher, Y. Ren

**Affiliations:** a University of Groningen, University Medical Center of Groningen, Department of Orthodontics, Hanzeplein 1, 9700 RB, Groningen, the Netherlands; b University of Groningen, University Medical Center Groningen, Department of Biomedical Engineering, Antonius Deusinglaan 1, 9713 AV, Groningen, the Netherlands

**Keywords:** Probiotic bacteria, Pathogenic bacteria, Reactive oxygen species, Infrared-spectroscopy, X-ray photoelectron spectroscopy

## Abstract

Bacteria are frequently used in industrial processes and nutrient supplementation to restore a healthy human microflora, but use of live bacteria is often troublesome. Here, we hypothesize that bacterially-derived carbon-quantum-dots obtained through pyrolytic carbonization inherit physico-chemical properties from probiotic and pathogenic source-bacteria. Carbon-quantum-dots carbonized at reaction-temperatures below 200 ​°C had negligible quantum-yields, while temperatures above 220 ​°C yielded poor water-suspendability. Fourier-transform infrared-spectroscopy demonstrated preservation of amide absorption bands in carbon-quantum-dots derived at intermediate temperatures. X-ray photoelectron-spectroscopy indicated that the at%N in carbon-quantum-dots increased with increasing amounts of protein in source-bacterial surfaces. Carbonization transformed hydrocarbon-like bacterial surface compounds into heterocyclic aromatic-carbon structures, evidenced by a broad infrared absorption band (920-900 ​cm^−1^) and the presence of carbon in C–C functionalities of carbon-quantum-dots. The chemical composition of bacterially-derived carbon-quantum-dots could be explained by the degradation temperatures of main bacterial cell surface compounds. All carbon-quantum-dots generated reactive-oxygen-species, most notably those derived from probiotic lactobacilli, carrying a high amount of surface protein. Concluding, amide functionalities in carbon-quantum-dots are inherited from surface proteins of source-bacteria, controlling reactive-oxygen-species generation. This paves the way for applications of bacterially-derived carbon-quantum-dots in which reactive-oxygen-species generation is essential, instead of hard-to-use live bacteria, such as in food supplementation or probiotic-assisted antibiotic therapy.

## Highlights

• Pyrolytic carbonization of bacteria between 200°C and 220°C yields water-suspendable CQDs.
• Bacterially-derived CQDs inherit amide functionalities from bacterial cell surface proteins.
• Hydrocarbon-like bacterial surface compounds give heterocyclic aromatic-carbon structures in bacterially-derived CQDs.
• Bacterially-derived CQDs possess graphitic nitrogen.
• Zeta potentials of CQDs relate with nitrogen occurrence in CQDs.

## Graphical abstract

**Figure:**
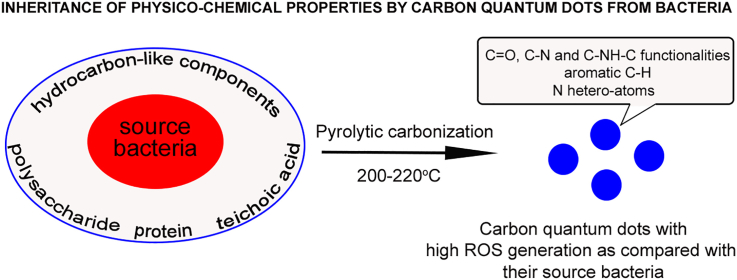

## 1 Introduction

Carbon quantum dots (CQDs) are nanoparticles possessing extremely small sizes, typically less than 10 ​nm. CQDs are currently considered for a variety of applications, ranging from biosensors [1], bio-imaging [2], drug delivery [3] and cancer therapy [4] to bacterial infection control [5]. CQDs can be synthesized “bottom-up” [6] from small precursor molecules, but also “top-down” [7] by breaking down large carbon-rich materials as a carbon source. CQDs synthesized from natural carbon sources, such as leaves [8], paper [9], honey [10], flour [11] or bacteria [12] can bear special properties due to differences in the inheritance of hetero-atoms or specific chemical functionalities from their sources [7,13,14]. This inheritance can be due to non-carbon atoms occurring in natural carbon sources that after carbonization, replace a carbon atom in the molecular structure of carbon dots. Typically, these hetero-atoms include nitrogen, phosphorus or sulphur. Similarities in infrared absorption spectra between gentamicin sulfate and CQDs derived through calcination of gentamicin sulfate demonstrated inheritance of antibacterial chemical functionalities from gentamicin [6]. Nitrogen-doped CQDs, hydrothermally synthesized from a bis-quaternary ammonium salt, demonstrated similar antibacterial activity as quaternary ammonium salts in solution [15].

Bacteria are nowadays considered as friend or foe depending on where they occur. Bacteria are used in green, eco-friendly applications, such as bioreactors [16], bio-fuel production [17], and biosensors [18]. Probiotic bacteria are increasingly used in nutrient supplementation to restore a healthy human microflora [19] or combat infectious pathogens in combination with antibiotic therapy [20] to which many bacterial pathogens have become resistant (probiotic-assisted antibiotic therapy). Often however, benefits of friendly bacteria cannot be optimally exploited due to their susceptibility to strong light conditions, extreme pH values such as encountered by probiotic bacteria in the gastrointestinal tract after oral administration, (self-produced) toxins in bioreactors and biosensors or inadvertent killing during antibiotic therapy intended to kill infectious pathogens [21,22].

Considering the similarities oberserved between gentamicin sulfate [6] and a bis-quaternary ammonium salt [15] and CQDs derived from them, we hypothesize that CQDs pyrolytically synthesized from different probiotic or pathogenic bacterial strains inherit physico-chemical properties from their source bacteria. Therefore, we here aim to find bacterial carbonization temperatures for which this hypothesis is true and explain the composition of bacterially derived CQDs based on degradation temperatures of main bacterial cell surface coumpounds. Six bacterial strains were included as natural carbon sources, i.e. three probiotic bacterial strains (*Bifidobacterium breve*, *Lactobacillus fermentum*, *Lactobacillus acidophilus*) and three human pathogens (*Escherichia coli*, *Klebsiella pneumoniae*, *Pseudomonas aeruginosa*). UV–vis absorption spectra and quantum yields of the CQD synthesized will be determined, while infrared absorption spectra, elemental surface compositions and zeta potentials will be compared for CQDs and their source bacteria. Generation of reactive oxygen species (ROS) is one of the trade-marks of probiotic bacteria [19] and hence their ROS generation was compared with those of bacterially derived CQDs. When proven true, our hypothesis will facilitate the use of CQDs in food supplementation or probiotic-assisted antibiotic therapy instead of hard-to-use live bacteria. Bacterially-derived CQDs can be prepared during manufacturing and can be administered without suffering the difficulties of live bacteria after oral administration in reaching their intestinal target site in a viable state due to the acidity of the gastro-intestinal tract or by antibiotics during probiotic-assisted antibiotic therapy.

## 2 Materials and methods

### 2.1 Materials

Reinforced Clostridial Medium (RCM) was obtained from Becton & Dickinson. Tryptone Soya Broth (TSB) and Brain Heart Infusion (BHI) were obtained from OXOID. De Man Rogosa and Sharpe (MRS), dipotassium hydrogen phosphate (purity >99.0%), potassium dihydrogen phosphate (purity >99.0%), NaOH (purity 99.0%) and KBr (purity 99.0%) were obtained from Merck KGaA. Quinine sulfate (USP testing specifications) and ethane (purity 99.99%) were obtained from Sigma. 2′,7′-dichlorofluorescein diacetate (DCFDA) was obtained from Invitrogen.

### 2.2 Bacterial growth and harvesting

All strains except *K. pneumoniae*-1 were purchased from the American Type Culture Collection (ATCC, USA). *K. pneumoniae*-1 is a human clinical isolate. All bacterial strains were kept as a frozen stock. For experiments, bacteria were thawed and grown on agar plates. Probiotic *B. breve* ATCC 15700 was grown on a RCM agar plate under anaerobic conditions (85% N_2_, 10% H_2_, 5% CO_2_). Probiotic *L*. *acidophilus* ATCC 4356 and *L. fermentum* ATCC 23271 were grown on MRS agar plates under 5% CO_2_. The three pathogenic strains (*E. coli* ATCC 25922, *K. pneumoniae*-1 and *P. aeruginosa* ATCC 10345) were grown on blood agar plates under aerobic conditions. All strains were grown at 37 ​°C.

For each strain, one colony was taken from an agar plate and inoculated in 10 ​mL RCM (for *B. breve*), MRS broth (for *L. acidophilus* and *L. fermentum*), BHI for *E. coli* or TSB for *K. pneumoniae* and *P. aeruginosa*). Incubation was done for 24 ​h under the appropriate conditions, as listed above. This preculture was transferred to 200 ​mL growth medium and incubated for 18 ​h, i.e. upon entering early stationary phase. Use of early stationary phase bacteria is generally preferred because possible small variations in culture conditions will have less impact while viability is still high [23]. Bacterial cultures were harvested by centrifugation for 5 ​min at 5000 ​*g*, washed twice with demineralized water and collected in ceramic crucibles for further synthesis of CQDs. For bacterial characterization, washed bacterial pellets were collected in 15 ​mL centrifuge tubes, frozen in liquid nitrogen, and subsequently freeze-dried (Edwards freeze dryer, Richmond Scientific, UK) for Fourier-Transform InfraRed spectroscopy (FTIR, Cary 600 series, Agilent Technologies, USA) and X-ray Photoelectron Spectroscopy (XPS, S-probe, Surface Science Instruments, Mountain View, CA, USA). For zeta potential measurements, bacterial pellets were suspended in 10 ​mM potassium phosphate buffer by sonication for 30 ​s, while cooling in an ice/water bath. Bacteria were enumerated using a Bürker-Türk counting chamber and re-suspended in 10 ​mM potassium phosphate buffer (pH 7.4) to a concentration of 1.0 ​× ​10^8^/mL. Suspensions were routinely spread on agar plates (RCM for *B. breve*, MRS broth for *L. acidophilus* and *L. fermentum*, BHI for *E. coli* or TSB for *K. pneumoniae* and *P. aeruginosa*) to verify that the viability of the suspended bacteria was >98%. This ensures that a concentration of 1.0 ​× ​10^8^ bacteria per mL as used here, corresponds with >0.98 ​× ​10^8^ ​CFU/mL (Colony Forming Units).

### 2.3 Synthesis of bacterial derived CQDs

For the synthesis of CQDs, bacteria were heated in crucibles at a rate of 5 ​°C/min in air to different reaction temperatures ranging between 160 ​°C and 240 ​°C. After reaching the target temperature, bacteria were kept at the reaction temperature for 2 ​h. After 2 ​h, the crucibles containing the reaction product were cooled down to room temperature taking approximately 3 ​h and reaction products were dispersed in demineralized water and sonicated for 2 ​h. For purification and obtaining suspensions with mono-disperse CQDs, suspensions were filtered through a 0.22 ​μm polyvinylidene difluoride filter membrane, followed by dialysis against demineralized water (cut-off 1 ​kDa) for 48 ​h under stirring at room temperature. For storage, the CQD suspension was freeze-dried and CQDs were stored at 4 ​°C.

### 2.4 Optical features and quantum yields of bacterially derived CQDs

Fluorescence emission spectra from the CQDs were measured using a spectrofluorometer (Jasco FP-8300, Japan). UV–vis absorption spectra of bacterially derived CQDs were obtained using a 5082 PerkinElmer Lambda 2 spectrophotometer. For both types of spectroscopy, CQDs were suspended in water in quartz cuvettes to a UV–vis absorbance below 0.1. Fluorescence emission and UV–vis absorption spectra were also taken of quinine sulfate (0.1 ​M ​H_2_SO_4_) as a standard for the calculation of the quantum yield (QY) of bacterially derived CQDs [2], according to

(1) $${\text{Φ}}_{x}={\text{Φ}}_{st}\left(\frac{K_{x}}{K_{st}}\right){\left(\frac{\eta_{x}}{\eta_{st}}\right)}^{2}$$

in which Φ_x_ and Φ_st_ are the quantum yields of the CQDs to be determined and the quinine sulfate standard (54%), respectively, K_x_ and K_st_ are the slopes of the absorbance as a function of the integrated fluorescence calculated by linear regression analysis and η_x_ and η_st_ are the refractive indices of the CQDs suspensions and the standard solution, respectively. Experiments were carried out for CQDs synthesized at different reaction temperatures.

### 2.5 Characterization of CQDs and bacteria

CQDs were imaged using cryo Transmission Electron Microscopy (TEM). CQD suspensions were prepared by adding 1 ​mg of CQDs into 1 ​mL of demineralized water under sonication. The CQD suspension was applied to a perforated carbon-coated foil (Quantifoil 3.5/1) and vitrified in ethane using a Vitrobot (FEI, The Netherlands). CQDs were imaged with a FEI Tecnai T20 electron microscope operating at 200 ​keV. Three TEM images were used to measure the diameters of the CQDs, comprising a total of 260 CQDs for each type of CQD. Diameters were plotted in histograms with 0.4 ​nm binning size. Average diameters and standard deviations of the distributions were calculated by fitting a log-normal function to the data.

Zeta potentials of the CQDs (500 ​μg/mL) and bacteria (1.0 ​× ​10^8^/mL) were measured in 10 ​mM potassium phosphate buffer at pH 7.4 representing physiological conditions, using a ZetaSizer (Malvern Instruments, UK) at 25 ​°C.

FTIR spectra were taken from freeze-dried bacteria and CQDs by pressing bacteria or CQDs mixed with KBr into a tablet. A KBr-tablet without bacteria or CQDs was used as a background. FTIR spectra were recorded over the wavenumber range of 4000 to 400 ​cm^−1^ with a resolution of 4 ​cm^−1^. 32 scans were taken and averaged for each type of CQD.

Elemental surface compositions of freeze-dried bacteria and CQDs were determined using XPS. To this end, freeze-dried bacteria were pressed in small stainless steel cups, while CQDs were deposited on gold-coated glass slides and put in the XPS chamber. For measurements, the chamber was brought to a vacuum of 10^−9^ ​Pa. X-rays (10 ​kV, 22 ​mA) were produced using an aluminum anode and had a spot size of 250 ​× ​1000 ​μm. Wide scans were made of the overall spectrum in the binding energy range of 1–1200 ​eV at low resolution, setting the C_1s_ photoelectron binding energy peak at 284.8 ​eV [24]. High resolution, narrow scans were recorded over a 20 ​eV binding energy range of the C_1s_ and N_1s_ photoelectron binding energy peaks. The area under each peak in overall spectra, after background subtraction, was used for the calculation of peak intensities, yielding elemental surface concentrations. Narrow scans were decomposed into different, Gaussian peak components employing fixed positions of the binding energies of the different components [24] and full-widths-at-half-maximum of 1.4 ​eV and 1.6 ​eV for C_1s_ and N_1s_ peak components, respectively (Casa Software Ltd, Teignmouth, UK).

### 2.6 Generation of reactive oxygen species by bacterially derived CQDs

Generation of Reactive Oxygen Species (ROS) is common to many types of CQDs [25,26] and is one of the trade-marks of probiotic bacteria [19]. Hence, the functionality of the physico-chemical inheritance of bacterially derived CQDs with respect to ROS generation of CQDs derived from the different probiotic and pathogenic bacterial strains was compared with the ROS generation of the source bacteria. ROS generation by CQDs was determined according to a published method [27], based on oxidation of non-fluorescent 2′,7′-dichlorofluorescein (DCFH) to highly fluorescent 2′,7′-dichlorofluorescein (DCF). DCFH was prepared by hydrolysis of 10 ​mg/mL 2′,7′-dichlorofluorescein diacetate (DCFDA) dissolved in dimethylsulfoxide for 30 ​min in the presence of 0.01 ​M NaOH in the dark at room temperature. Next, the mixture was neutralized with potassium phosphate buffer pH 7.4 to obtain a final concentration of 10 ​μg/mL DCFH. Freshly prepared CQDs suspensions (200 ​μL, 500 ​μg/mL) were put in 96-wells plates, 10 ​μL DCFH was added and the mixture was kept in the dark for 1 ​h at 37 ​°C. After 1 ​h, fluorescence intensities due to oxidation of DCFH into DCF as a measure of ROS generation, were determined at excitation and emission wavelengths of 500 and 535 ​nm, respectively. Fluorescence intensity of DCFH dissolved in potassium phosphate buffer without CQDs amounted 3.7 ​± ​0.1 (arbitrary units). ROS generation by bacteria was determined similarly by mixing 200 ​μL bacterial suspension (1 ​× ​10^8^ bacteria/mL) in 10 ​mM potassium phosphate buffer with 10 ​μL DCFDA (10 ​μg/mL in 10 ​mM potassium phosphate buffer) in a 96-wells plate. The plates were kept in the dark for 1 ​h at 37 ​°C, and DCFDA was subsequently hydrolysed and oxidized by ROS to yield fluorescent DCF. DCF was quantitated as described above as a measure of bacterial ROS production. Fluorescence intensity of DCFDA only dissolved in 10 ​mM potassium phosphate buffer without bacteria amounted 0.2 ​± ​12 (arbitrary units).

### 2.7 Statistical analyses

Data were evaluated for statistical differences using a Student's *t*-test. Relationships between physico-chemical properties of different source bacteria and bacterially derived CQDs were subjected to a Pearson's rank correlation test and linear regression, depending on the measured parameters involved in order to reveal increases or decreases between the data (GraphPad Software, USA).

## 3 Results

### 3.1 Optical features, quantum yields and diameters of bacterially derived CQDs

First, specific optical features of the bacterially derived CQDs synthesized at different reaction temperatures were determined.

**Fig. 1:**
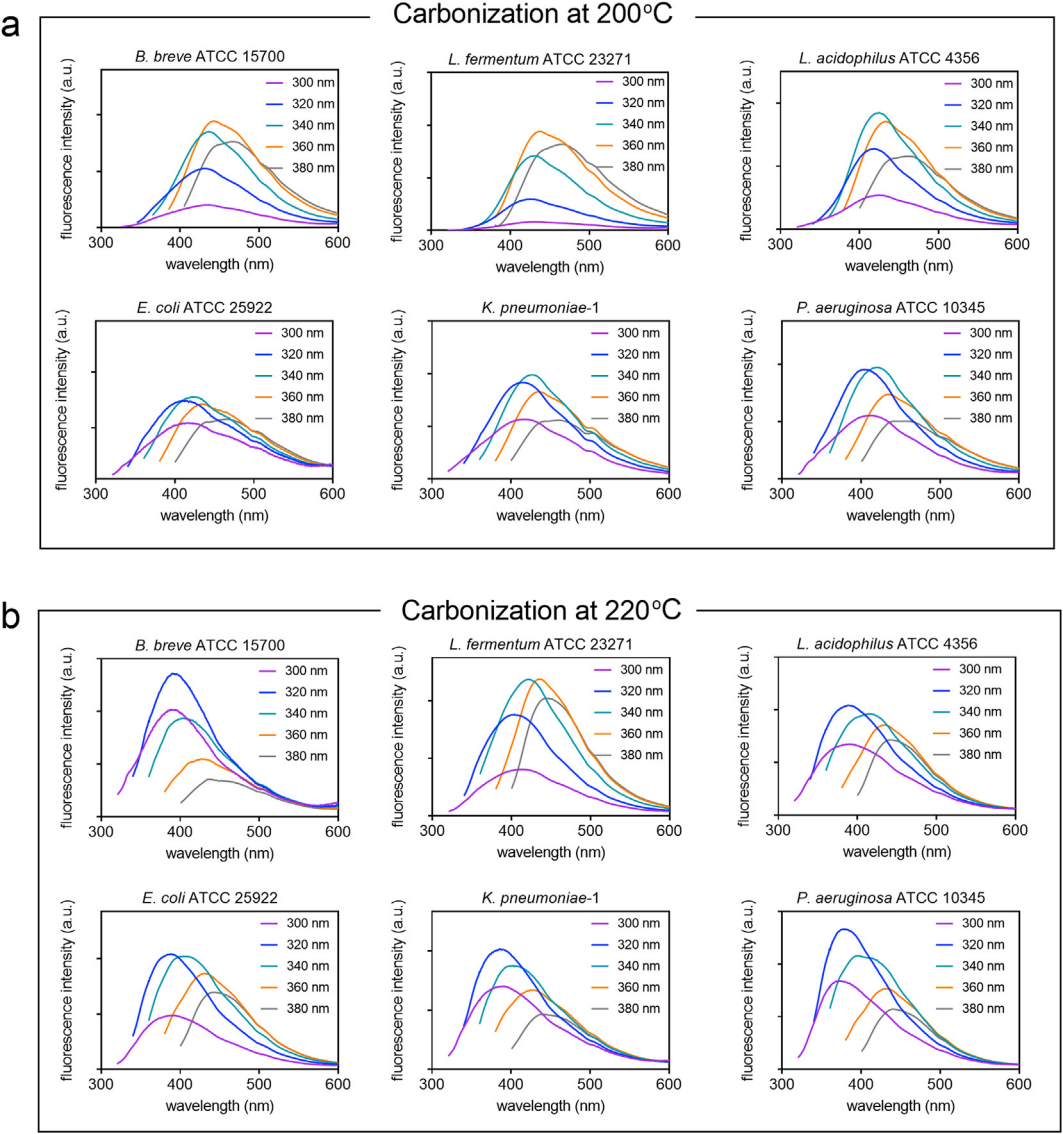
Fluorescence emission spectra of CQDs pyrolytically synthesized from different strains of probiotic and pathogenic source bacteria taken at different excitation wavelengths. **(a)** Carbonization at a reaction temperature of 200 ​°C. **(b)** Carbonization at a reaction temperature of 220 ​°C.

Fluorescence emission intensities of all CQDs strongly depended on wavenumber and differed across the different source-strains used (Fig. 1). For CQDs carbonized at 220 ​°C emission peaks appeared at lower wavelengths than for CQDs carbonized at 200 ​°C.

**Fig. 2:**
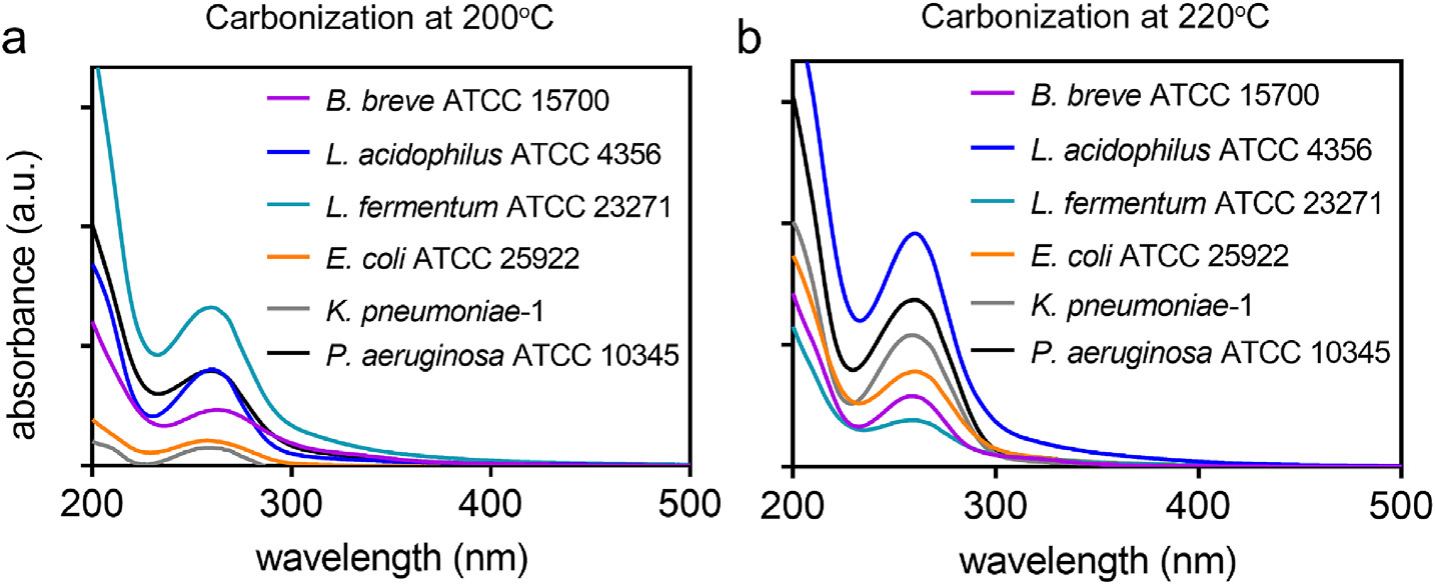
UV–vis absorption spectra of pyrolytically synthesized CQDs from different strains of probiotic or pathogenic source bacteria. **(a)** Carbonization at a reaction temperature of 200 ​°C. **(b)** Carbonization at a reaction temperature of 220 ​°C.

UV–vis absorption spectra of all bacterially derived CQDs, had a strong absorption band around 260 ​nm due to the π-π∗ transitions of conjugated C=C bonds in CQDs [28] (Fig. 2).

Next, fluorescence quantum yields were calculated as illustrated in Fig. S1 and summarized in Table 1 for the different bacterial strains used as carbon sources and different reaction temperatures.

**Table 1:** Quantum yields of CQDs pyrolytically synthesized at different reaction temperatures from different strains of probiotic and pathogenic source bacteria. Quantum yields were expressed relative to quinine sulfate as a standard. Data in bold represent the reaction temperature at which the quantum yield was maximal for each type of CQD (∗p < 0.05, Student's *t*-test *versus* the maximal value). All data are expressed as means ​± ​standard deviations over duplicate experiments over two batches of CQDs synthesized from two different bacterial cultures.

Reaction temperature (^o^C)	Quantum yields (%)					
	*B. breve* ATCC 15700	*L. fermentum* ATCC 23271	*L. acidophilus* ATCC 4356	*E. coli* ATCC 25922	*K. pneumoniae*-1	*P. aeruginosa* ATCC 10345
240	NS^a^	NS	NS	NS	NS	NS
220	0.5 ​± ​0.3	**1.4 ​± ​0.9**	**1.0 ​± ​0.2**	**1.5 ​± ​0.2**	**1.2 ​± ​0.2**	**1.9 ​± ​0.5**
200	**1.0 ​± ​0.2**	0.7 ​± ​0.3	0.5 ​± ​0.1	0.1 ​± ​0.0∗	0.2 ​± ​0.1∗	0.4 ​± ​0.1∗
180	0.2 ​± ​0.1∗	0.6 ​± ​0.1	0.3 ​± ​0.1∗	0.1 ​± ​0.0∗	0.0 ​± ​0.0∗	0.1 ​± ​0.1∗
160	0.2 ​± ​0.1∗	0.3 ​± ​0.1	0.1 ​± ​0.1∗	0.1 ​± ​0.1∗	0.0 ​± ​0.0∗	0.0 ​± ​0.0∗

a NS indicates CQDs obtained were not suspendable in water.

Quantum yields were negligible (<0.5%) up to reaction temperatures of 180 ​°C, indicating absence of CQD formation. Further increases in reaction temperature yielded higher quantum yields. Reaction temperatures above 220 ​°C resulted in poorly water-suspendable CQDs [28,29] that could not be used in further experiments. On average, quantum yields where higher upon pyrolytic carbonization at 220 ​°C than at 200 ​°C, while at 220 ​°C quantum yields of CQDs derived from pathogenic strains were slightly higher than when derived from probiotic strains. Accordingly, all data in the remainder of this article refer to CQDs pyrolytically synthesized at 200 ​°C and 220 ​°C.

**Fig. 3:**
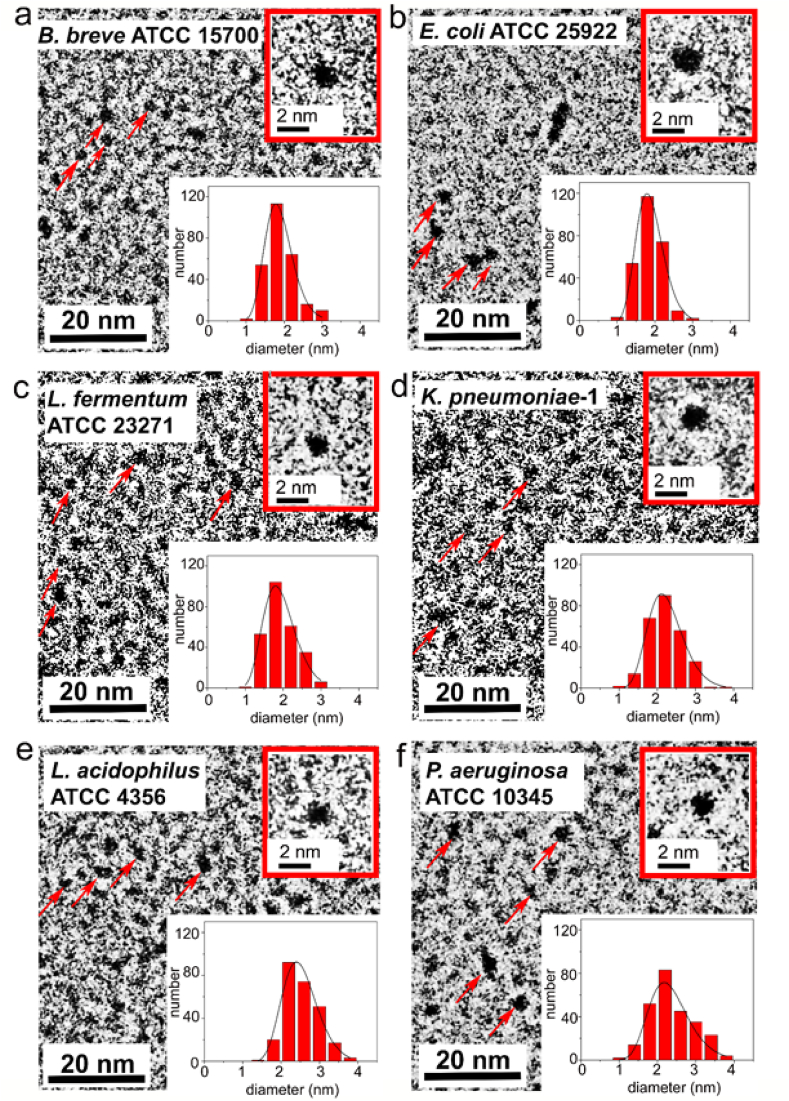
TEM micrographs and diameter distributions of CQDs pyrolytically synthesized from different strains of probiotic and pathogenic source bacteria at a reaction temperature of 220 ​°C. CQDs are indicated by red arrows in each micrograph. **(a)***B. breve* (diameter 1.9 ​± ​0.4 ​nm, as averaged over 260 different CQDs (equally divided over two batches of CQDs synthesized from different bacterial cultures) with ± indicating the standard deviation). **(b)***E. coli* (1.9 ​± ​0.4 ​nm). **(c)***L. fermentum* (1.9 ​± ​0.4 ​nm). **(d)***K. pneumoniae* (2.2 ​± ​0.5 ​nm). **(e)***L. acidophilus* (2.5 ​± ​0.5 ​nm). **(f)***P. aeruginosa* (2.3 ​± ​0.5 ​nm). Average diameters and standard deviations were calculated by fitting a log-normal curve (solid lines) to the data (binning size 0.4 ​nm).

Cryo-TEM micrographs (Fig. 3) indicated that all CQDs possessed a spherical shape, regardless of the bacterial strain used for carbonization. TEM micrographs after digital enlargement, were applied to derive the diameters of the CQDs synthesized, which ranged between 1.9 ​nm and 2.5 ​nm across CQDs derived from different source strains and all possessed a relatively small standard deviation as calculated by fitting a log-normal curve to the diameter distribution. This attests to the efficacy of the filtration and dialysis procedures applied to obtain mono-disperse CQDs.

### 3.2 Surface chemistry of source bacteria - FTIR and XPS

**Fig. 4:**
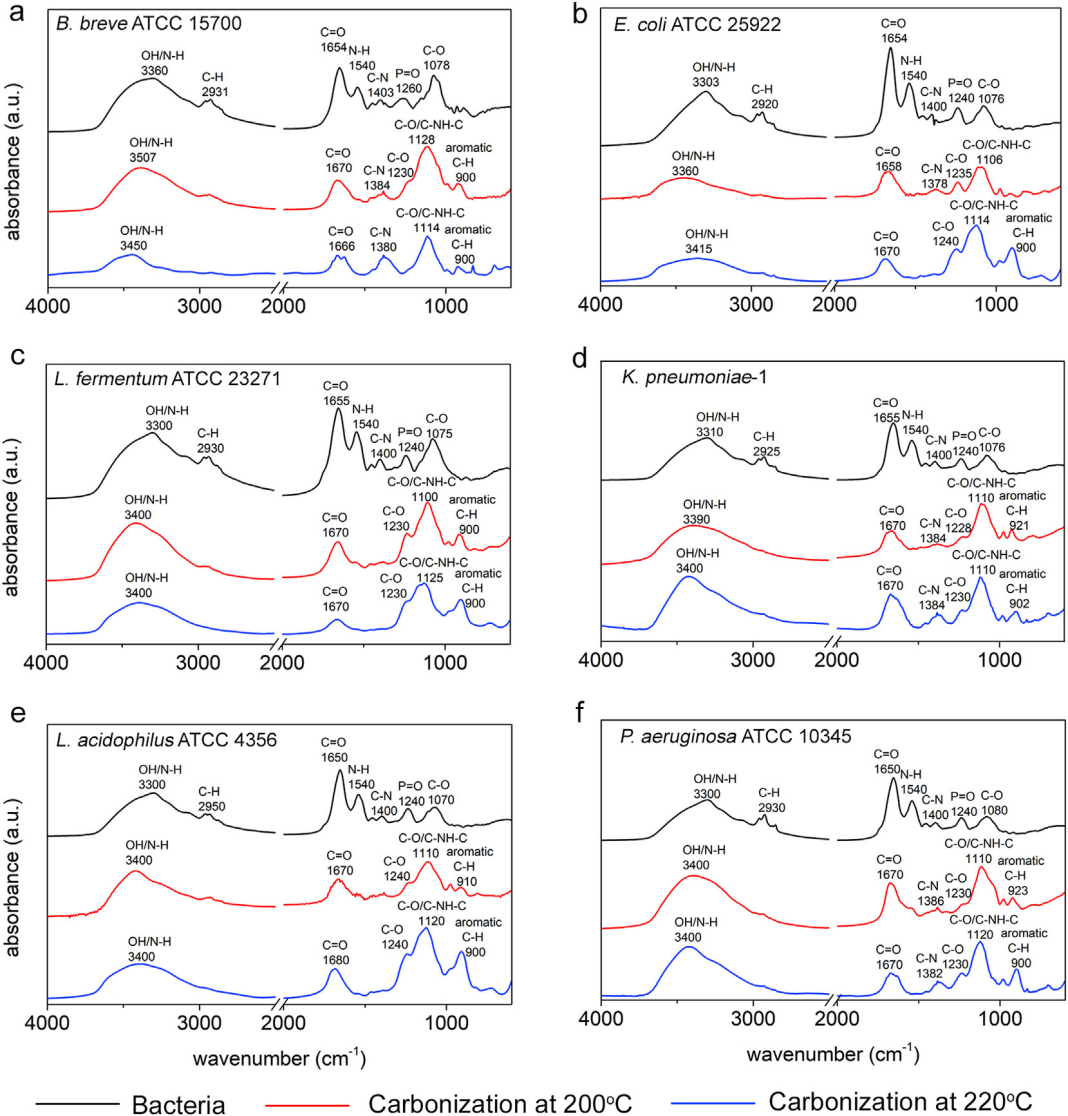
Examples of FTIR spectra of the different probiotic and pathogenic bacterial strains and CQDs derived from them by pyrolytic carbonization at reaction temperatures of 200 ​°C and 220 ​°C. **(a)***B. breve* and *B. breve* derived CQDs. **(b)***E. coli* and *E. coli* derived CQDs. **(c)***L. fermentum* and *L. fermentum* derived CQDs. **(d)***K. pneumoniae* and *K. pneumoniae* derived CQDs. **(e)***L. acidophilus* and *L. acidophilus* derived CQDs. **(f)***P. aeruginosa* and *P. aeruginosa* derived CQDs. FTIR spectra have been taken in duplicate over two batches of CQDs synthesized from different bacterial cultures, showing spectra with identical absorption bands.

FTIR spectra of the different bacterial strains used are presented in Fig. 4. FTIR spectra of the different source bacteria all possessed absorption bands at similar wavenumbers that only differed in absorbance, as also demonstrated in other studies [[30], [31], [32], [33]].

Absorption bands between 3400 and 3300 ​cm^−1^ were attributed to O–H and N–H stretching, while absorption bands between 2950 and 2920 ​cm^−1^ were attributed to C–H stretching vibrations. Protein associated bands appeared around 1650 ​cm^−1^ (Amide I) and 1540 ​cm^−1^ (Amide II) were attributed to stretching vibrations of C=O and bending vibrations of N–H, respectively [30,31]. Stretching vibrations of C–N in proteins occurred around 1400 ​cm^−1^. The absorption bands between 1260 ​cm^−1^ and 1240 ​cm^−1^ (PI) were assigned to stretching vibrations of P=O from phosphodiester of nucleic acids and teichoic acids. Absorption bands between 1080 ​cm^−1^ and 1070 ​cm^−1^ (PII) originated from C–O–C stretching in bacterial polysaccharides [32,33].

**Fig. 5:**
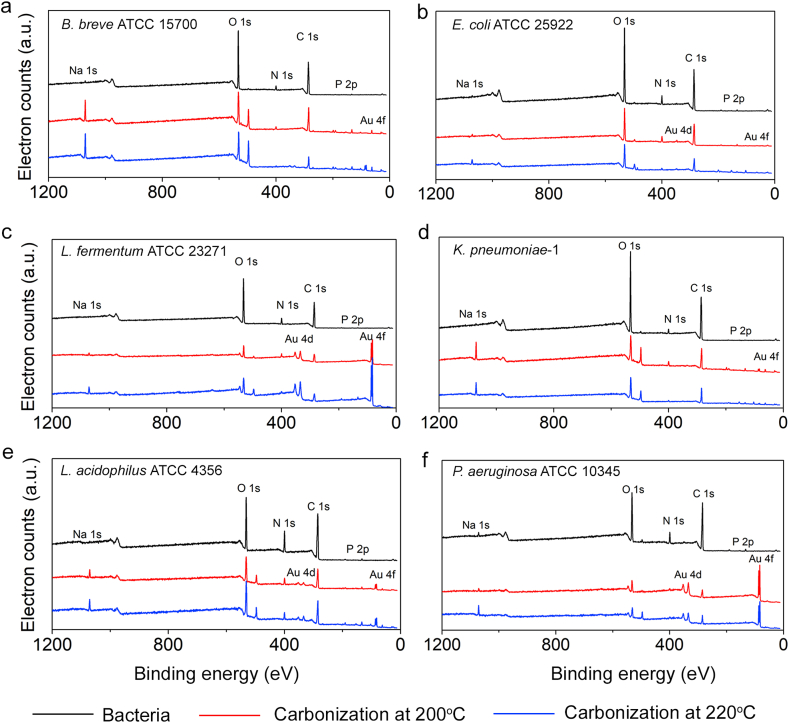
Examples of XPS wide-scan binding energy spectra of the different probiotic and pathogenic bacterial strains and CQDs derived from them by pyrolytic carbonization at reaction temperatures of 200 ​°C and 220 ​°C. **(a)***B. breve* and *B. breve* derived CQDs. **(b)***E. coli* and *E. coli* derived CQDs. **(c)***L. fermentum* and *L. fermentum* derived CQDs. **(d)***K. pneumoniae* and *K. pneumoniae* derived CQDs. **(e)***L. acidophilus* and *L. acidophilus* derived CQDs. **(f)***P. aeruginosa* and *P. aeruginosa* derived CQDs. Note the Au_4f_ peak originates from the gold-coated glass slides to which the CQDs were attached. XPS spectra have been taken in duplicate over two batches of CQDs synthesized from different bacterial cultures, showing spectra with electron binding energy peaks at identical positions.

XPS wide-scan binding energy spectra of the different bacterial strains used are presented in Fig. 5. Electron binding energy peaks of the different source bacteria all appeared at the same binding energies, mainly demonstrating the presence of carbon, oxygen, nitrogen and a number of minor elements amongst which phosphorus, as also demonstrated in other studies [[33], [34], [35], [36]].

In an interpretative, generalized model of XPS elemental compositions of bacterial cell surfaces, the bacterial cell surface is considered to be composed of protein (Pr), peptidoglycan (Pg), teichoic acid (Ta), polysaccharide (Ps) and hydrocarbon-like compounds (Hc) [37]. Based on the theoretical elemental composition ratios of these compounds measured using XPS, elemental surface concentration ratios with respect to carbon can be expressed in terms of the fractions of Pr, Pg, Ta, Ps and Hc according to Ref. [37].

(2) N⁄C ​= ​0.270 x C_Pr_+0.200 x C_Pg_

(3) O⁄C ​= ​0.320 x C_Pr_+0.500 x C_Pg_+1.200 x C_Ta_+0.833 x C_Ps_

(4) P⁄C ​= ​0.170 x C_Ta_

(5) 1 ​= ​C_Pr_ ​+ ​C_Pg_ ​+ ​C_Ta_ ​+ ​C_Ps_ ​+ ​C_Hc_

In which C_i_ represents the fraction of carbon associated with each compound. The fractions calculated using Eqs. (2), (3), (4), (5), can be converted in to weight fractions employing the carbon concentration in each model component [33,37]. Since the peptidoglycan layer is positioned in the interior of what is generally called the cell wall and covered by a layer of proteins, polysaccharides and teichoic acid, the surface concentration of Pg is assumed to be zero in these calculations [37]. Table 2 indicates different amounts of protein, hydrocarbon, teichoic acid and polysaccharide on the surfaces of the different source strains. As compared with the pathogenic strains involved, higher amounts of surface protein were found on the surfaces of the probiotic strains as a group, especially when considering both lactobacillus strains.

**Table 2:** Biochemical surface composition of the probiotic and pathogenic bacterial strains involved in this study, derived from XPS data empoying Eqs. (2), (3), (4), (5). All data are expressed as means ​± ​standard deviations over duplicate experiments with different bacterial cultures.

Bacterial strain	Biochemical composition of bacterial cell surfaces (wt%)			
	Protein	Hydro-carbon	Teichoic acid	Polysaccharide
*B. breve* ATCC 15700	21.7 ​± ​2.1	16.0 ​± ​1.8	9.3 ​± ​0.0	53.0 ​± ​0.3
*L. fermentum* ATCC 23271	33.9 ​± ​1.0	8.4 ​± ​1.5	9.9 ​± ​1.1	47.7 ​± ​0.6
*L. acidophilus* ATCC 4356	66.5 ​± ​3.8	9.0 ​± ​3.7	14.4 ​± ​1.0	10.2 ​± ​0.9
*E. coli* ATCC 25922	30.2 ​± ​1.3	6.7 ​± ​1.2	16.7 ​± ​0.0	46.4 ​± ​2.4
*K. pneumoniae*-1	16.0 ​± ​0.8	19.7 ​± ​2.5	6.3 ​± ​4.2	58.0 ​± ​7.4
*P. aeruginosa* ATCC 10345	41.0 ​± ​3.5	19.7 ​± ​7.1	21.5 ​± ​2.0	17.7 ​± ​5.5

### 3.3 Surface chemistry of CQD-inheritance from their source bacteria

FTIR spectra of CQDs obtained from pyrolytic carbonization of bacteria, beared both similarities and dissimilarities with bacterial FTIR spectra (see Fig. 4). The C–H stretching band between 2950 and 2920 ​cm^−1^ had disappeared at the highest reaction temperature. The C=O stretching band around 1650 ​cm^−1^ (Amide I) had broadened upon carbonization to become overlapped with the N–H bending band around 1540 ​cm^−1^ (Amide II), as compared with spectra of their source bacteria. The C–N stretching vibrations at 1380 ​cm^−1^ had disappeared in CQDs derived from *E. coli*, *L. fermentum* and *L. acidophilus*, but remained observable in *B. breve*, *K. pneumoniae* and *P. aeruginosa* derived CQDs. After carbonization, a broad band developed between 1260 ​cm^−1^ and 1100 ​cm^−1^. The P=O/C–O stretching bands between 1260 ​cm^−1^ and 1240 ​cm^−1^ (PI) visible in spectra of source bacteria, had decreased strongly as well, but remained observable as a weak shoulder of this broad band around 1230 ​cm^−1^ due to C–O stretching [38,39]. At the low wavenumber side of the same broad band, the PII bands in spectra of source bacteria due to polysaccharides remained observable in CQDs between 1128 ​cm^−1^ and 1100 ​cm^−1^.

**Fig. 6:**
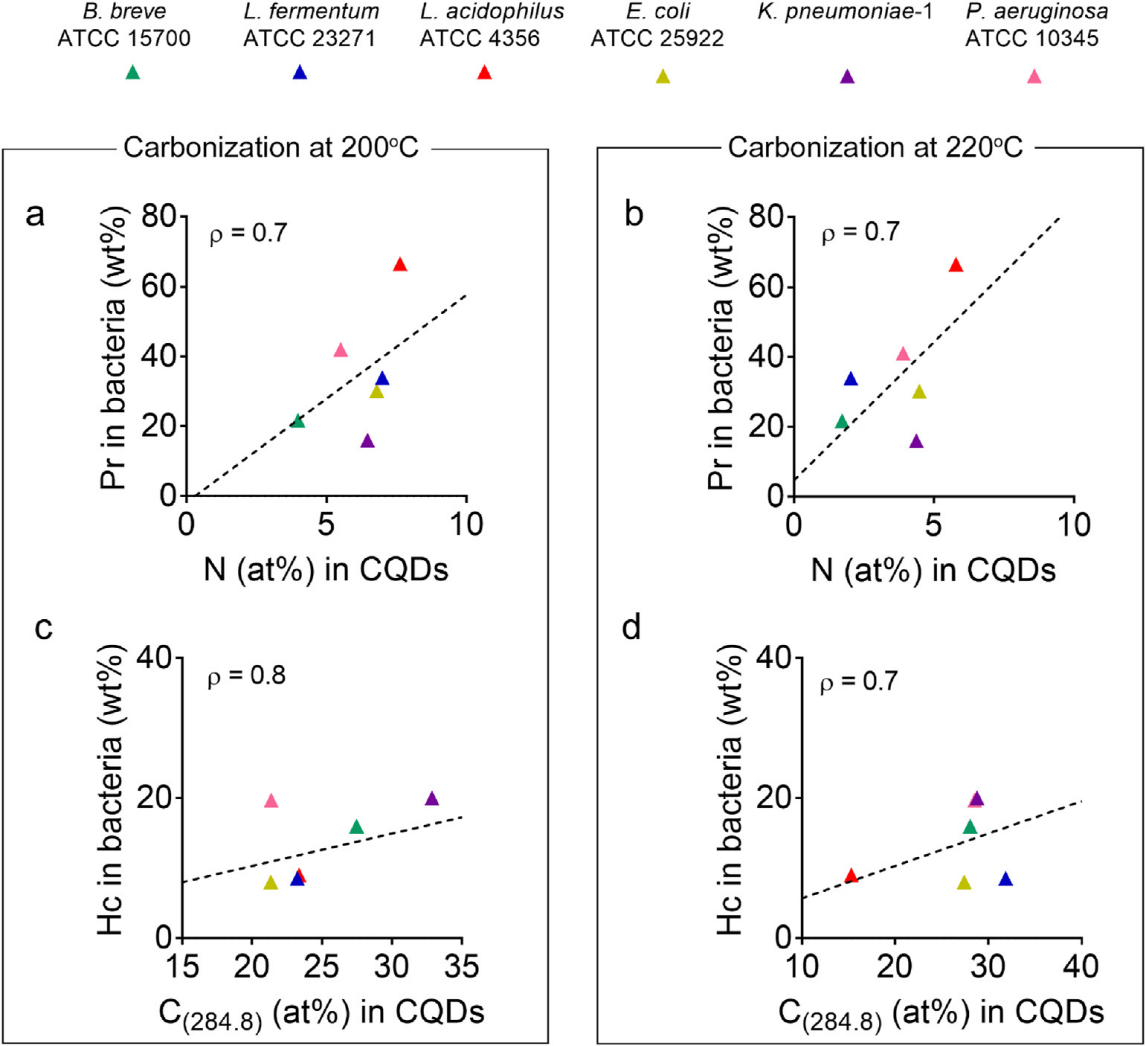
Nitrogen and C–C functionalities in CQDs inherited from bacterial cell surface components after carbonization at different reaction temperatures. **(a)** Bacterial cell surface protein as a function of the prevalence of nitrogen in CQDs synthesized at 200 ​°C. **(b)** Same as (a) but now for CQDs synthesized at 220 ​°C. **(c)** Bacterial cell surface hydrocarbon-like compounds as a function of the prevalence of carbon involved in C–C functionalities in CQDs synthesized at 200 ​°C. **(d)** Same as (c) but now for CQDs synthesized at 220 ​°C. Pearson's rank correlation tests have been done on all graphs to discern increases or decreases between the data (dotted lines), with rank correlation coefficients ρ indicated in the graphs. All data represent averages of duplicate experiments over two batches of CQDs synthesized from different bacterial cultures. Results from duplicate experiments had standard deviations in bacterial surface protein and hydrocarbon-like compounds of 1.9 ​wt% and 3.9 ​wt%, respectively, while for CQDs standard deviations amounted 1.0 ​at% and 6.6 ​at% for the prevalence of N and carbon involved in C–C functionalities, respectively.

The nitrogen content in CQDs increased with an increasing amount of protein in the source bacteria, regardless of the reaction temperature (Fig. 6a and b). This suggests that nitrogen in CQDs is inherited from bacterial protein. Combined with the preservation of the amide bands in the FTIR spectra of bacterially derived CQDs (Fig. 4), it can be concluded that proteinaceous structures have been partly preserved upon carbonization of source bacteria. We could not find similar evidence of preservation of polysaccharide-like structures upon carbonization.

Fig. 6c and d presents the relation between the amount of hydrocarbon-like compounds in the surface of source bacteria (Table 2) according to Eqs. (2) to (5) with the prevalence of carbon in C–C functionalities in CQDs derived from decomposition of narrow scans of the C_1s_ binding energy spectra (Fig. S2). The increase in the percentage carbon involved in C–C bonds in CQDs with the amount of hydrocarbon-like compounds in the source bacterial surfaces in combination with the development of a new FTIR absorption band between 920 ​cm^−1^ and 900 ​cm^−1^ (Fig. 4) due to aromatic C–H bending [40], suggests that aromatic C–H structures in CQDs are inherited from carbonized bacterial cell surface hydrocarbon-like compounds.

**Fig. 7:**
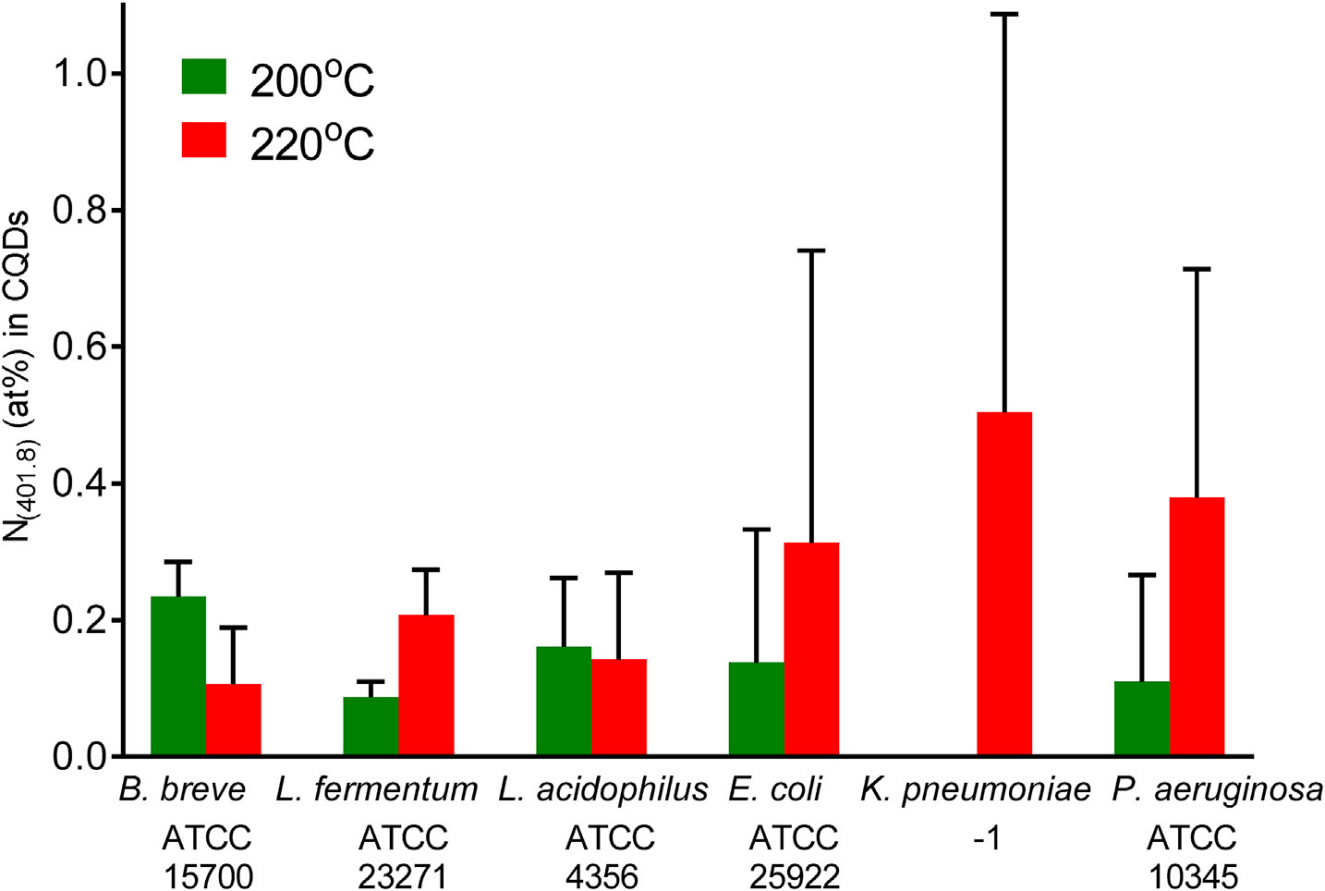
The prevalence of graphitic nitrogen (N_1s_ binding energy 401.8 ​eV [41]) in bacterially derived CQDs after synthesis at different reaction temperatures of 200 ​°C and 220 ​°C. All data are expressed as means over duplicate experiments with CQDs synthesized from different bacterial cultures.

The occurrence of nitrogen with a N_1s_ binding energy component at 401.8 ​eV in CQDs (Fig. S3) is indicative of the presence of graphitic nitrogen [41]. The prevalence of graphitic nitrogen in CQDs (Fig. 7) is generally lower when bacteria were carbonized at 200 ​°C than when carbonized at 220 ​°C. This conclusion is supported by the observation of aromatic C–H bending in FTIR spectra of bacterially derived CQDs (Fig. 4), of which also nitrogen hetero-atoms can be part.

### 3.4 Zeta potentials and ROS generation of bacterially derived CQD as related with their chemical inheritance

**Fig. 8:**
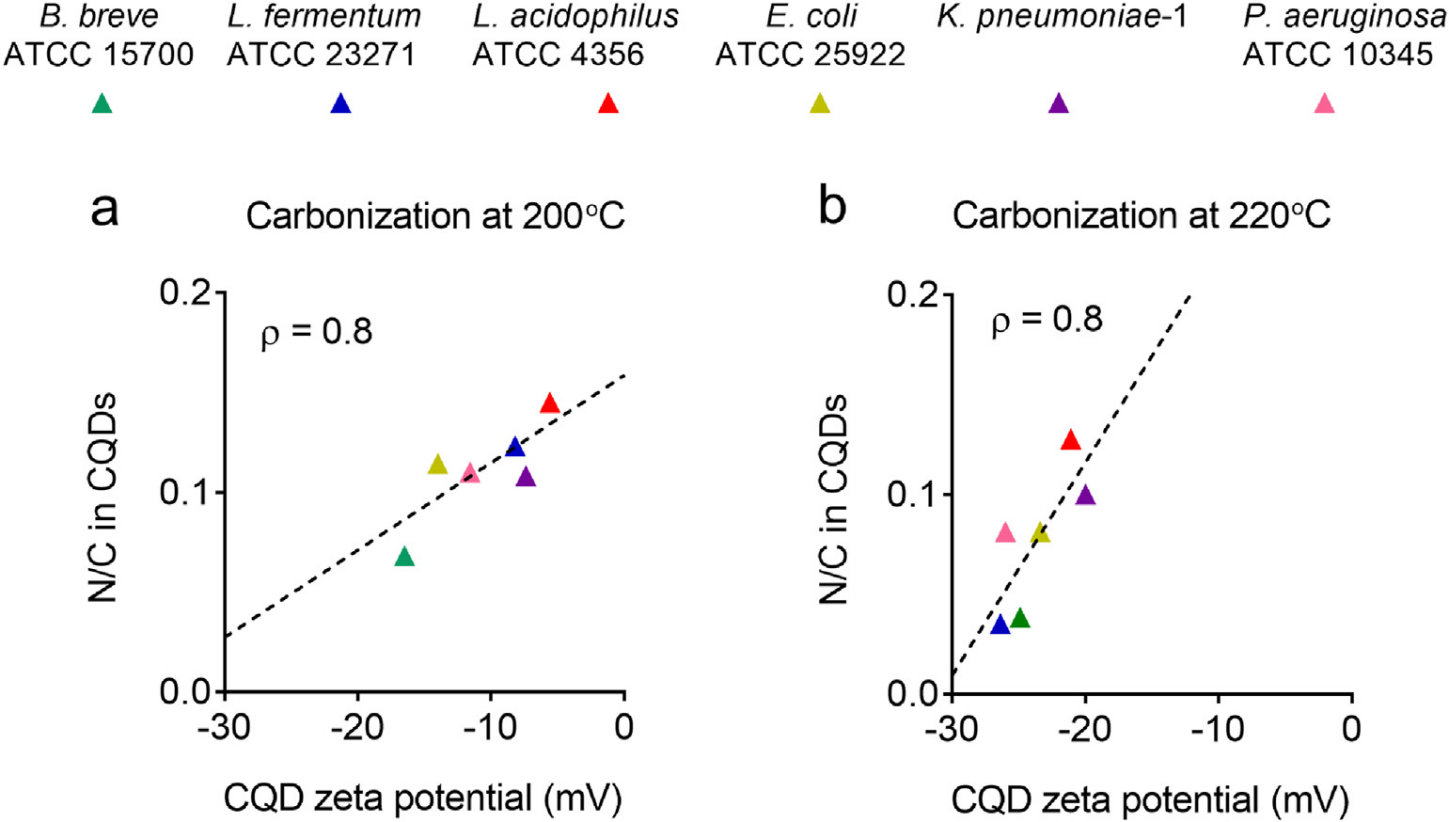
Role of inherited nitrogen in the zeta potentials of CQD inherited from bacterial cell surfaces after carbonization at different reaction temperatures. **(a)** N/C elemental concentration ratios of bacterially derived CQDs as a function of zeta potentials at pH 7.4 after synthesis at 200 ​°C. **(b)** Same as (a) but now after synthesis at 220 ​°C. Pearson's rank correlation tests have been done on all graphs to discern increases or decreases between the data (dotted lines), with rank correlation coefficients ρ indicated in the graphs. Zeta potentials represent averages over triplicate experiments with CQDs synthesized from different bacterial cultures, while XPS data represent duplicate experiments. Results from replicate experiments had standard deviations in N/C of 0.01 and in zeta potentials of 1.3 ​mV.

Inherited nitrogen played a determining role in the zeta potentials of all bacterially derived CQDs. Increasing elemental concentration ratios of nitrogen in CQDs related with less negative zeta potentials (Fig. 8), reflecting the role of amide groups as a positive charge carrier in bacterially derived CQDs.

ROS generation by source bacteria was just above the detection limit of the method applied. All bacterially derived CQDs generated ROS in 100-fold higher amounts than their source bacteria (Table 3). ROS generation by bacterially derived CQDs did not depend in a systematic way on reaction temperature (see also Table 3). *B. breve* derived CQDs generated significantly less ROS (p ​< ​0.05, Student's *t*-test) than CQDs derived from pathogenic bacteria and both lactobacillus strains. On average however, CQDs derived from lactobacillus strains generated two-fold more ROS (p ​< ​0.05, Student's *t*-test) than the average ROS generation by CQDs derived from pathogenic bacteria.

**Table 3:** Generation of reactive oxygen species by source bacteria and bacterially derived CQDs after synthesis at different reaction temperatures, measured in 10 ​mM potassium phosphate buffer at pH 7.4, expressed as a fluorescence intensity. All data are expressed as means ​± ​standard deviations over triplicate experiments with different CQDs.

Source strain	Fluorescence Intensity by bacteria (a.u)	Carbonization temperature (°C)	Fluorescence Intensity by CQDs (a.u)
*B. breve* ATCC 15700	0.3 ​± ​0.0	200	16.3 ​± ​0.4
		220	6.3 ​± ​0.3
*L. fermentum* ATCC 23271	0.3 ​± ​0.0	200	88.5 ​± ​4.7
		220	98.3 ​± ​1.4
*L. acidophilus* ATCC 4356	0.4 ​± ​0.1	200	51.7 ​± ​0.6
		220	40.0 ​± ​1.5
*E. coli* ATCC 25922	0.3 ​± ​0.0	200	48.5 ​± ​0.0
		220	41.5 ​± ​0.6
*K. pneumoniae*-1	0.3 ​± ​0.0	200	31.7 ​± ​1.2
		220	28.4 ​± ​1.3
*P. aeruginosa* ATCC 10345	0.3 ​± ​0.0	200	36.8 ​± ​1.3
		220	40.4 ​± ​1.0

## 4 Discussion

In this article, we demonstrate that CQDs derived from pyrolytically carbonized source bacteria, inherit amide groups from bacterial surface proteins, while hydrocarbon-like bacterial surface compounds are carbonized into heterocyclic aromatic-carbon structures, including graphitic nitrogen.

The quantum yields of CQDs synthesized at reaction temperatures below 200 ​°C were extremely low, while reaction temperatures above 220 ​°C resulted in poorly water-suspendable CQDs that could not be used in further experiments. Accordingly, all results in this article pertain to reaction temperatures of 200 ​°C or 220 ​°C. Poor suspendability in water of CQDs synthesized through pyrolysis at high reaction temperatures has been reported before [28] and is due to “over-carbonization”, implying thermally-induced degradation of hydrophilic moieties necessary for suspending CQDs in water. Candidate hydrophilic moieties for thermally-induced degradation around 200 ​°C to 220 ​°C, known to convey hydrophilicity to bacterial cell surfaces [42], are polysaccharides. Thermal gravimetric analyses of bacterial polysaccharides have shown that polysaccharides loose water between 40 ​°C and 180 ​°C [43], while degradation starts between 160 ​°C and 180 ​°C and is completed between 260 ​°C and 290 ​°C with exceptions going up to around 450 ​°C, depending the microbial origin of the polysaccharide [[43], [44], [45]]. Degradation temperatures of polysaccharides therewith explain why carbonization at reaction temperatures above 220 ​°C yielded CQDs that were not suspendable in water, but also why no indications of inheritance of polysaccharides by CQDs from the source bacteria used in this study could be observed.

In the approach chosen, conclusions about physico-chemical inheritances by CQDs from source bacteria were qualitatively based on correspondences in FTIR absorption spectra of CQDs and source bacteria, while more quantitative conclusions were based on ranking of compositional data of CQDs and bacterial cell surfaces obtained from XPS analyses. The quantitatively-based approach assumes that the interior of different bacterial strains contributes less to differences in a possible inheritage by CQDs than bacterial cell surface components. The validity of this assumption was demonstrated in the past from series of comprehensive relations between compositional data of a wide variety of different bacterial strains obtained using FTIR and XPS data. FTIR has a depth of information encompassing an entire bacterial cell, while XPS pertains only to the outermost layer of bacterial cell surface components. Relations between these two fundamentally different sets of data were taken as proof that physico-chemically the main distinction between bacterial strains is confined predominantly to their surface [30].

Using XPS data, the outermost surface of the source bacteria was modeled as being composed of protein, polysaccharide and teichoic acid (Eqs. (2)-(5)) [37]. Thus derived surface compositions of freeze-dried bacteria explained important bacterial cell surface properties such as their hydrophobicity and surface charge. Polysaccharide-rich bacterial cell surfaces were more hydrophilic measured through two-phase partitioning than protein-rich surfaces [46]. Also, strains with protein-rich surfaces had higher iso-electric points measured through particulate microelectrophoresis than teichoic acid-rich bacterial cell surfaces [47]_._ Bacterial cell surface hydrophobicity and charge are both measured in an aqueous environment with fully hydrated bacteria, while oppositely the interpretative XPS model [37] employs data in a fully de-hydrated state. Therefore, the correspondence between these two sets of data was taken as evidence that bacterial cell surface XPS reflects cell surface composition relevant in a hydrated state [48], as common to the bacterial source strains used in this article. Importanly, the high protein contents in the surfaces of freeze-dried *L. fermentum* and *L. acidophilus* calculated from XPS data, reflect the crystalline protein layer that the surfaces of many lactobacillus strains possess [49].

Protein degradation products of pyrolytically carbonized bacteria were found in all CQDs, most notably the above two lactobacillus strains. Apart from loosing water upon heating, proteins change their conformation. Therewith the molecular environment of characteristic chemical groups in proteins changes with an impact on FTIR absorption spectra. Typically, the α-helix protein structure is transformed into a β-turn structure in proteins upon heating up to 70 ​°C [50], shifting and broadening the amide I band and reducing the amide II band [51], as can also be observed by comparing FTIR spectra of the CQDs pyrolytically synthesized here with those of their source bacteria. Protein degradation starts around 200 ​°C [45], leaving amide functionalities for incorporation in CQDs derived from carbonization of source bacteria. Effective degradation of amide functionalities through random scission of the C–N, C(O)–NH, C(O)–NH_2_, –NH_2_ and C(O)–OH bonds of the proteins, only occurs at much higher temperatures than applied here for pyrolytic carbonization, beginning at around 300 ​°C [52] and alluding full protein degradation at higher temperatures [45]. Degradation of lipids and other (hydrocarbon-like) compounds in microbial cell surfaces commence at temperatures as low as 116 ​°C up to 635 ​°C [45] depending on the strain considered. Degradation products of these bacterial cell surface compounds explain the formation of heterocyclic aromatic-carbon structures and the higher occurrence of graphitic nitrogen in CQDs synthesized through pyrolytic carbonization of bacteria at 220 ​°C as compared with 200 ​°C.

The chemical inheritage of bacterially derived CQDs from their source bacteria is interesting, but not sufficient to advocate use of these CQDs instead of live bacteria. To this end, also functional properties need to be inherited. Zeta potentials of bacterially derived CQDs depended on the inheritance of amide groups from the source bacteria. As also observed in bacteria, possession of more protein was accompanied by less negative zeta potentials [47,48]. Importantly, generation of reactive oxygen species was around 100-fold higher by CQDs than by their source bacteria, most notably for CQDs derived from *L. fermentum* and *L. acidophilus*, possessing extremely protein-rich surfaces. These observations make the inheritance of amide groups in bacterially derived CQDs essential in the maintenance of important functional properties of the source strains in bacterially derived CQDs.

## 5 Conclusions

Based on Fourier transform infrared and X-ray photoelectron spectroscopic analyses of CQDs we conclude that bacterially derived CQDs inherit important chemical groups from their source bacteria upon pyrolytic carbonization at reaction temperatures of 200 ​°C and 220 ​°C. Especially inheriting amide groups turned out to be essential for maintaining important characteristics of the source bacteria, such as their zeta potentials controlling an important part of their environmental interaction and the generation of ROS. However, it is likely that different bacterial strains and species than employed here, must be carbonized at different reaction temperatures to optimize the physico-chemical inheritance from source bacteria in bacterially derived CQDs.

The consequences of the inheritance observations in bacterially derived CQDs may be far reaching. Bacteria are used nowadays in many different applications and the use of live bacteria carries several problems. In general, bacteria may not only carry a health risk, but the use of bacteria requires culturing, storage of bacteria is not trivial and bacteria may not be able to survive industrial or environmental conditions, including those encountered by probiotic bacteria in the gastro-intestinal tract after oral administration. This study demonstrates that (1) the chemical composition of CQDs bears similarity with the surface composition of their source bacteria, provided carbonization is done at appropriate reaction temperatures, (2) functional properties, including superior ROS generation as compared with their source bacteria, accompany the chemical composition of CQDs in a similar fashion as in their source bacteria. Therewith this study paves the way for the use of CQDs as a substitute for the use of live bacteria in different applications, including application of probiotic bacteria in food supplementation or probiotic-assisted antibiotic therapy of bacterial infections. Therewith many of the problems associated with the use of live bacteria can be circumvented.

## CRediT author statement

Yanyan Wu: Conceptualization, Methodology, Investigation, Writing – original draft, Hao Wei: Investigation, Writing – review & editing, Henny C. van der Mei: Conceptualization, Methodology, Writing – review & editing, Supervision, Joop de Vries: Investigation, Writing – review & editing, Henk J. Busscher: Conceptualization, Methodology, Writing – review & editing, Supervision, Yijin Ren: Conceptualization, Writing – review & editing, Supervision, Funding acquisition.

## Data availability

Data are available on request.

## Declaration of competing interest

The authors declare the following financial interests/personal relationships which may be considered as potential competing interests: HJB is also director of a consulting company SASA BV. Opinions and assertions contained herein are those of the authors and are not construed as necessarily representing views of the funding organization or their respective employer(s).
