# Supplementary material for: Inheritance of physico-chemical properties and ROS generation by carbon quantum dots derived from pyrolytically carbonized bacterial sources
Source: Mater Today Bio. 2021 Oct 15;12:100151. doi: 10.1016/j.mtbio.2021.100151 (PMC8554632; doi:10.1016/j.mtbio.2021.100151)
Supplement: Multimedia component 1 [file mmc1.docx]

Supporting Information

Inheritance of physico-chemical properties and

ROS generation by carbon quantum dots derived

from pyrolytically carbonized bacterial sources

*Yanyan Wu,^a^ Hao Wei,^b^ Henny C. van der Mei, ^b*^ Joop de Vries, ^b^ Henk J. Busscher,^b^ Yijin Ren^a^*

^a^ University of Groningen and University Medical Center of Groningen Department of Orthodontics

Hanzeplein 1, 9700 RB, Groningen, The Netherlands

^b^ University of Groningen and University Medical Center Groningen Department of Biomedical Engineering

Antonius Deusinglaan 1, 9713 AV, Groningen, The Netherlands

*Corresponding author: h.c.van.der.mei@umcg.nl

Department of Biomedical Engineering-FB40

University Medical Center Groningen

Antonius Deusinglaan 1

9713 AV Groningen

The Netherlands

Tel +31 50 3616096

**Figure S1.** Examples of integrated fluorescence intensity as a function of absorbance of CQDs pyrolytically synthesized from different strains of source bacteria and quinine sulfate as a standard for deriving the quantum yields of the carbon dots. Synthesis was done at different reaction temperatures ranging from 160°C to 220°C.

**
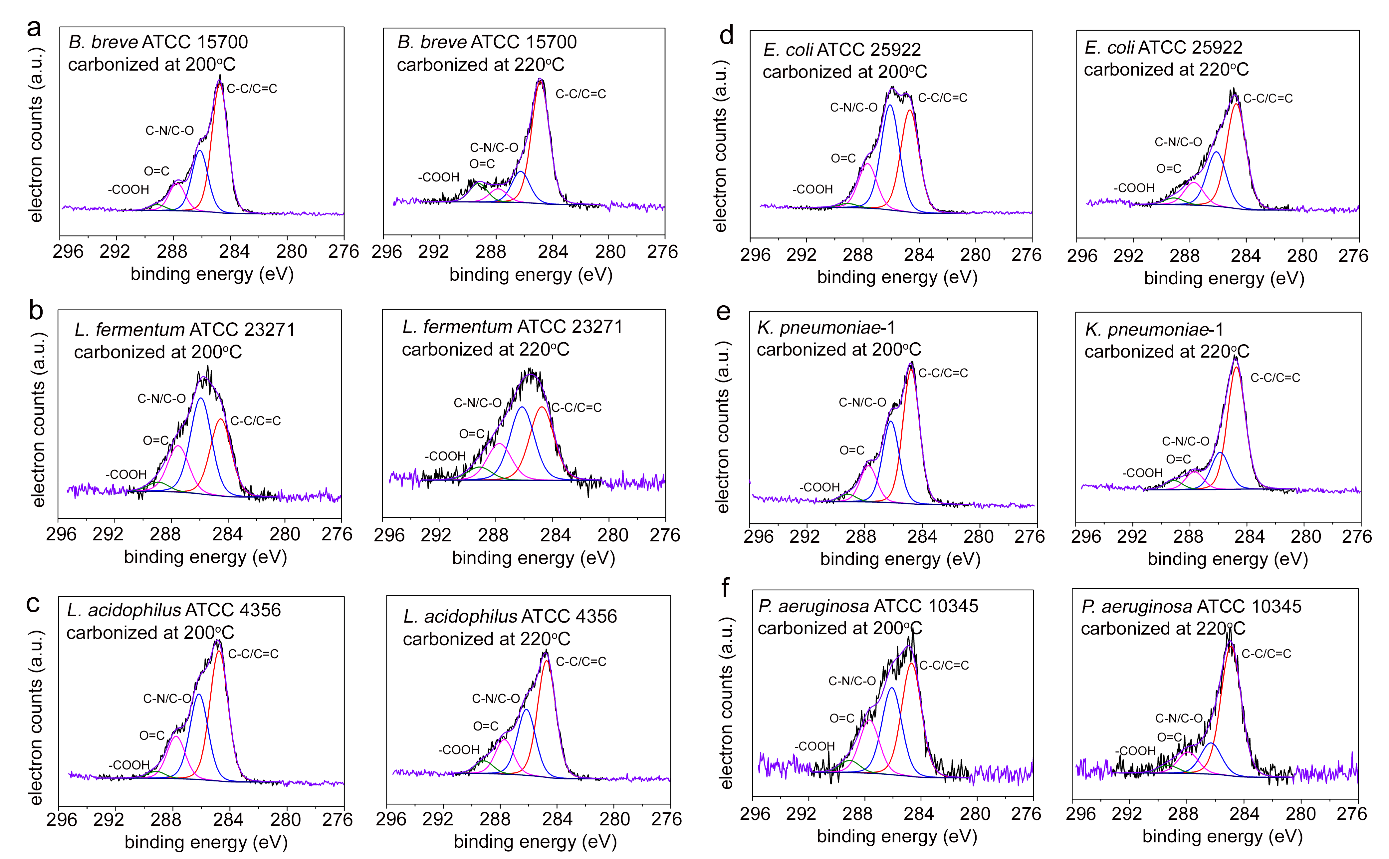
**

**Figure S2.** Examples of narrow-scans of C_1s_ electron binding energy spectra of bacterially derived CQDs (reaction temperatures 200°C and 220°C) and their decomposition into four Gaussian components at 284.8, 286.3, 287.8 and 289.2 eV. (a) *B. breve* CQDs. (b) *L. fermentum* CQDs. (c) *L. acidophilus* CQDs. (d) *E. coli* CQDs. (e) *K. pneumoniae* CQDs*.* (f) *P. aeruginosa* CQDs.

**
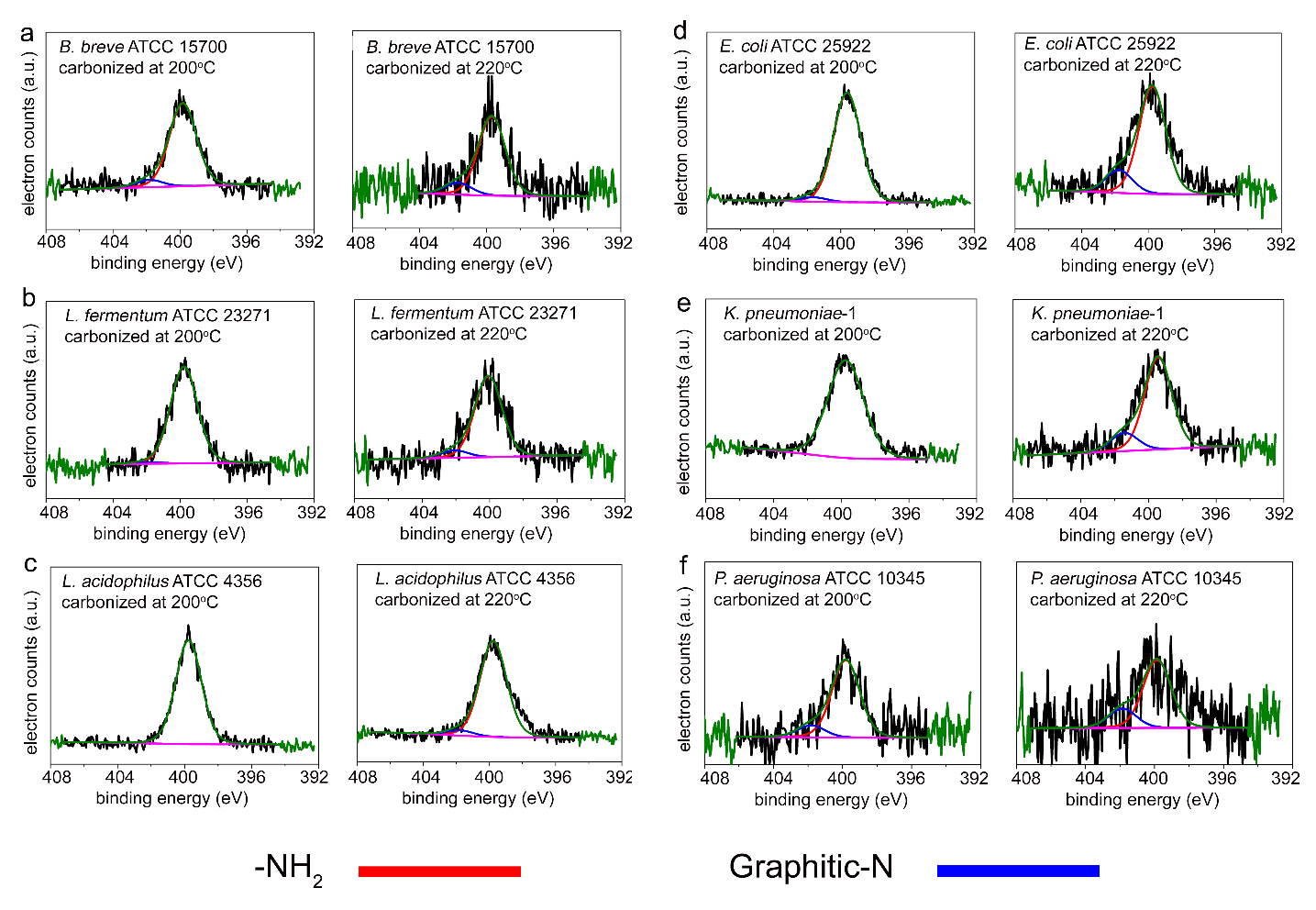
**

**Figure S3.** Examples of narrow-scans of N_1s_ electron binding energy spectra of bacterially derived CQDs (reaction temperatures 200°C and 220°C) and their decomposition into two Gaussian components at 399.8 (red) and 401.8 (blue) eV. (a) *B. breve* CQDs. (b) *L. fermentum* CQDs. (c) *L. acidophilus* CQDs. (d) *E. coli* CQDs. (e) *K. pneuminiae*-1 CQDs*.* (f) *P. aeruginosa* CQDs.
